# An Essay to Prove the Contagious Character of Malignant Cholera, with Brief Instructions for Its Prevention and Cure

**Published:** 1855-08

**Authors:** 


An essay to prove the Contagious character of Malignant Cholera, with brief instructions for its prevention and cure. By Bernard M. Byrne, M.D., Surgeon U.S Army. Second edition, with additional notes by the author. Philadelphia : Childs & Peterson. 1855.

The discovery of the cause, nature, cure and prevention of Epidemic Cholera. By M. L. Knapp, M.D., etc.

The first of these essays is a new edition of a pamphlet by the author, published in 1833.

The facts adduced in favor of Contagion are divided into two classes, general and particular. The general facts are :

1. Its power of progression.
2. The continuous line of posts by which it travels.
3. The irregularity of its progress.
4. The preference which it manifests for those routes in which there is the greatest intercourse.
5. The irregularity of its periods of increase and decline in the different places which it visits.

The particular facts consist of individual instances, in which the disease has been developed simultaneously with or subsequently to the arrival of persons afflicted with it. The author seems to us to leave the subject as much befogged as he found it. Among the extravagant assertions demanding for its belief a greater faith than ours, is the following : "If we estimate the proportion of attendants who actually die of cholera in the country, we shall find that in many places it exceeds five hundred to one of any other class of inhabitants."

Under the head of prevention, he holds the following language, which seems to us to be virtually yielding the question, or at least leaving to it the same uncertainty which attaches to all epidemics :

In order to guard against the general incursions of cholera, as well as against its individual attacks, it is necessary to be particularly mindful of two facts : one is, that the poison which produces this disease has its source in the human body *only;* the other, that this poison is, in a majority of cases, incapable of producing the disease, unless assisted by an exciting cause.

In reference to treatment the following is perhaps worthy of note:

The grand indication for the cure of cholera, in all its stages, is to restore the secretions; and to accomplish this there is no agent with which we are acquainted so powerful as mercury. It is impossible, therefore, that patients can go wrong in endeavoring, before the arrival of a physician, to bring their systems under the influence of this remedy; for it is one which will generally save life, if promptly and copiously employed in this stage of the disease.

The treatment in collapse differs materially from that which is employed in the other stages of cholera. Diffusible stimulants, large doses of calomel, and powerful astringent injections, are the remedies on which we must mainly rely. In this stage, blood-letting is fatal; and frictions of every description are useless, if not pernicious. Our principal aim should be, to raise the sinking powers of the system by stimulants, while we endeavor at the same time to equalize them by the exhibition of calomel.

The best stimulants for this purpose are, spirits of camphor, sulphuric ether, ammoniated alcohol, tincture of prickly ash, and the various species of ardent spirits.

Dr. Knapp assumes that the cause that produces cholera is an "error in the vital stimulus of food," and that that error consists in a "scarcity," that "*cholera is but a modified form of scorbutus*, or a young sister scourge of the same family, probably better expressed by calling it a hœmorrhagic termination or a manifestation of the dying phenomena of scorbutus." The remainder of the pamphlet is occupied in endeavoring to prove this hypothesis. J.

